# A New Crested Pterosaur from the Early Cretaceous of Spain: The First European Tapejarid (Pterodactyloidea: Azhdarchoidea)

**DOI:** 10.1371/journal.pone.0038900

**Published:** 2012-07-03

**Authors:** Romain Vullo, Jesús Marugán-Lobón, Alexander W. A. Kellner, Angela D. Buscalioni, Bernard Gomez, Montserrat de la Fuente, José J. Moratalla

**Affiliations:** 1 Laboratoire Géosciences Rennes, CNRS UMR 6118, Université de Rennes 1, Rennes, France; 2 Unidad de Paleontología, Departamento de Biología, Universidad Autónoma de Madrid, Madrid, Spain; 3 Laboratory of Systematics and Taphonomy of Fossil Vertebrates, Departamento de Geologia e Paleontologia, Universidade Federal do Rio de Janeiro, Museu Nacional, Rio de Janeiro, Brazil; 4 Laboratoire de Géologie de Lyon, Terre, Planètes, Environnement, CNRS UMR 5276, Université de Lyon 1, Villeurbanne, France; 5 Instituto Geológico y Minero de España, Museo Geominero, Madrid, Spain; College of the Holy Cross, United States of America

## Abstract

**Background:**

The Tapejaridae is a group of unusual toothless pterosaurs characterized by bizarre cranial crests. From a paleoecological point of view, frugivorous feeding habits have often been suggested for one of its included clades, the Tapejarinae. So far, the presence of these intriguing flying reptiles has been unambiguously documented from Early Cretaceous sites in China and Brazil, where pterosaur fossils are less rare and fragmentary than in similarly-aged European strata.

**Methodology/Principal Findings:**

*Europejara olcadesorum* gen. et sp. nov. is diagnosed by a unique combination of characters including an unusual caudally recurved dentary crest. It represents the oldest known member of Tapejaridae and the oldest known toothless pterosaur. The new taxon documents the earliest stage of the acquisition of this anatomical feature during the evolutionary history of the Pterodactyloidea. This innovation may have been linked to the development of new feeding strategies.

**Conclusion/Significance:**

The discovery of *Europejara* in the Barremian of the Iberian Peninsula reveals an earlier and broader global distribution of tapejarids, suggesting a Eurasian origin of this group. It adds to the poorly known pterosaur fauna of the Las Hoyas locality and contributes to a better understanding of the paleoecology of this Konservat-Lagerstätte. Finally, the significance of a probable contribution of tapejarine tapejarids to the early angiosperm dispersal is discussed.

## Introduction

In the Early Cretaceous (145–99 million years ago), a peak occurred in the morphological disparity of Pterosauria, suggesting that new ecological niches were exploited [1]–[4]. One of the most striking adaptations in the evolutionary history of several groups of pterosaurs is the development of distinctively crested skulls. The Tapejaridae [5], [6] is one of the groups that have appeared during this period and that have acquired these important ecomorphological innovations. This clade of pterodactyloids is represented by peculiar forms, which are subdivided into two groups: the long-faced and large Thalassodrominae and the short-faced and smaller Tapejarinae [6], [7]. Both bear well-developed cranial crests [6]–[8], making the tapejarids a bizarre-looking and enigmatic group of pterosaurs.

The subfamily Tapejarinae comprises the oldest known edentulous pterosaurs [9], [10]. It has been interpreted that the acquisition of a suite of ecomorphological novelties was key to develop the behavior and feeding strategies (i.e., frugivory) that helped tapejarines exploit unexplored ecological niches. Due to their short skull with a particular downturned rostrum and unusually shaped toothless beak, tapejarines have most consistently been suggested to have been seed and/or fruit eaters [9], [11], [12].

This group of peculiar pterodactyloids is uncommon in the fossil record, occurring mainly in some late Early Cretaceous (Aptian–Albian stages, 125–99 million years ago) fossil Konservat-Lagerstätten of Brazil (e.g. *Tapejara*, *Tupandactylus*) [7] and China (e.g. *Sinopterus*) [9]. In addition, a single jaw fragment from the early Late Cretaceous (Cenomanian stage, 99–93 million years ago) of Morocco has been tentatively referred to as an indeterminate tapejarid [6], [13], [14]. Despite the diversity and richness of the European Cretaceous pterosaur faunas [15]–[17], tapejarids have not been reported from this continent so far.

Here, we report an incomplete skull and lower jaw of a new tapejarid pterosaur, *Europejara olcadesorum* gen. et sp. nov., from the mid-Early Cretaceous (Barremian stage, 130–125 million years ago) La Huérguina Formation laminated limestone of Las Hoyas (Cuenca) in eastern Spain. This new taxon is the oldest known tapejarid and the first found in Europe, thus extending the stratigraphic and geographical distribution of the group. It differs from all other known tapejarids by bearing a well-developed caudally recurved sagittal crest (autapomorphy) on the lower jaw. *Europejara* sheds new light on the evolutionary history of the group based upon a new phylogenetic analysis, suggesting that the origin of these toothless pterosaurs took place in Eurasia near the beginning of the Early Cretaceous. The late Barremian age of the Las Hoyas beds [18] also makes *Europejara* the oldest hitherto known toothless pterosaur, slightly older than *Eopteranodon* from the earliest Aptian of the Yixian Formation, China [19]–[21]. As in some groups of non-avian theropod dinosaurs [22] and early birds [23], the acquisition of complete edentulism, which appears to have occurred independently twice [24] or more likely at least three times [20], [25], [26] during the evolutionary history of Pterosauria, could be correlated with the development of new feeding strategies [9]. Within the Azhdarchoidea (i.e. the clade grouping chaoyangopterids, tapejarids, and azhdarchids), the complete tooth loss as well as some other morphological adaptations observed in the jaws of tapejarines could indicate herbivorous habits and be linked to the early angiosperm diversification [11]. However, such an assumption is based essentially on rostral morphology and lacks direct support from the fossil record that could be conclusively provided by gut contents only. In addition, although a co-existence in time and space of tapejarids and early flowering plants can be qualitatively observed, the fossil record and distribution of these pterosaurs are too scarce to provide any convincing quantitative evidence for an interaction between both groups. In this context, the hypothesis arguing that tapejarine tapejarids were frugivorous pterosaurs and significant long-distance dispersal vectors during the first radiation of flowering plants is here briefly discussed on the basis of the data available. Given the widespread geographical distribution and the particular adaptations of tapejarids (e.g. for possible frugivory), we discuss why studies should consider them a distinct component part of the Cretaceous Terrestrial Revolution [27].

## Materials and Methods

### Institutional Abbreviations

AMNH: American Museum of Natural History, New York, USA; BSP: Bayerische Staatssammlung für Paläontologie, Munich, Germany; CPCA: Centro de Pesquisas Paleontológicas da Chapada do Araripe, Crato, Brazil; GMN: Geological Museum of Nanjing, Nanjing, China; IVPP: Institute of Vertebrate Paleontology and Paleoanthropology, Beijing, China; MCCM-LH: Las Hoyas collection of the Museo de las Ciencias de Castilla–La Mancha, Cuenca, Spain.

### The Las Hoyas Fossil Site

The Las Hoyas subbasin is located in the Serranía de Cuenca, southwestern Iberian ranges, eastern Spain [28]. The sequence including the Las Hoyas fossiliferous deposits is composed of laminated limestone and rare marlstone levels (La Huérguina Formation, upper Barremian) [18]. These deposits were produced in the context of a continental subtropical, wet and forested environment overlying a low-relief karstic terrain [29].

The Konservat-Lagerstätte of Las Hoyas has yielded a rich floral and faunal continental assemblage [18], [30], [31]. The Las Hoyas biota mainly consists of obligate aquatic organisms (e.g. osteichthyan fishes, decapod crustaceans, belostomid insects, charophytes, the aquatic plant *Montsechia*) [29], [31]. The amphibious forms (i.e. crocodiliforms, turtles, and lissamphibians) are much less abundant, and the facultative ones (i.e. terrestrial/arboreal forms such as insects, lizards and basal birds) are rather rare. Large animals such as dinosaurs (e.g. *Concavenator*) [32] are exceptional in the assemblage, and correspond to the incidental ecological category [33]. Pterosaurs are rare in the Las Hoyas assemblage, with only a few teeth indicating the presence of a rather large ornithocheirid ( =  anhanguerid) and a small basal istiodactylid [34]. The terrestrial macroflora is dominated by conifers (Cheirolepidiaceae) and ferns (Matoniacea and Schizaeaceae), although angiosperm remains are present and relatively diverse [31].

Among the most significant taphonomic features of the vertebrate specimens of this site are their articulation and preservation (e.g. mineralization of soft tissue) [32], [35]. However, the Las Hoyas beds also contain numerous isolated vertebrate remains, such as teeth, ribs, feathers and fish scales.

### Phylogenetic Analysis

In order to access the phylogenetic position of *Europejara olcadesorum* gen. et sp. nov., we performed a phylogenetic analysis using PAUP 4.0b10 for Microsoft Windows [36] using the TBR heuristic searches performed using maximum parsimony (see Appendix S1 for character list and data matrix). Characters were given equal weight and treated unordered (ACCTRAN setting). This analysis is based on previous studies (e.g. [1], [20], [24]–[26], [37]). The search conducted by PAUP, with *Ornithosuchus longidens* and *Herrerasaurus ischigualastensis* as outgroups, produced 135 equally parsimonious trees (205 steps; consistency index = 0.7561; retention index = 0.8538; rescaled consistency index = 0.6456), from which a strict consensus cladogram was obtained.

Although a discussion of previous phylogenetic analyses is not the scope of this paper, we did also score *Europejara olcadesorum* in the phylogenetic analysis published by Andres & Ji [20] and obtained a similar result.

### Nomenclatural Acts

The electronic version of this document does not represent a published work according to the International Code of Zoological Nomenclature (ICZN), and hence the nomenclatural acts contained in the electronic version are not available under that Code from the electronic edition. Therefore, a separate edition of this document was produced by a method that assures numerous identical and durable copies, and those copies were simultaneously obtainable (from the publication date noted on the first page of this article) for the purpose of providing a public and permanent scientific record, in accordance with Article 8.1 of the Code. The separate print-only edition is available on request from PLoS by sending a request to PLoS ONE, 1160 Battery Street, Suite 100, San Francisco, CA 94111, USA along with a check for $10 (to cover printing and postage) payable to “Public Library of Science”.

In addition, this published work and the nomenclatural acts it contains have been registered in ZooBank, the proposed online registration system for the ICZN. The ZooBank LSIDs (Life Science Identifiers) can be resolved and the associated information viewed through any standard web browser by appending the LSID to the prefix “http://zoobank.org/”. The LSID for this publication is: urn:lsid:zoobank.org:pub:60DF87C8-E4CA-4021-877F-0EF6FA78B1BC.

## Results

### Systematic Paleontology

**Systematic hierarchy:**

Pterosauria Kaup, 1834

Pterodactyloidea Plieninger, 1901

Azhdarchoidea Nessov, 1984 (*sensu* [24])

Tapejaridae Kellner, 1989

Tapejarinae Kellner, 1989 (*sensu* [7])

*Europejara olcadesorum* gen. et sp. nov.

**ZooBank Life Science Identifier (LSID) for genus.**

urn:lsid:zoobank.org:act:69302E4D-F4BF-472F-9C72-2105BE44A227.

**ZooBank LSID for species.**

urn:lsid:zoobank.org:act:AED2386E-16C6-40B1-9E75-1EE06579AD95.

#### Etymology

Genus name formed by the combination of Europe and *Tapejara*, the internal specifier of the Tapejarinae [5], [7]; species named after the Olcades, Celtiberians who were the first inhabitants of the Cuenca region.

#### Holotype

**Figure 1:**
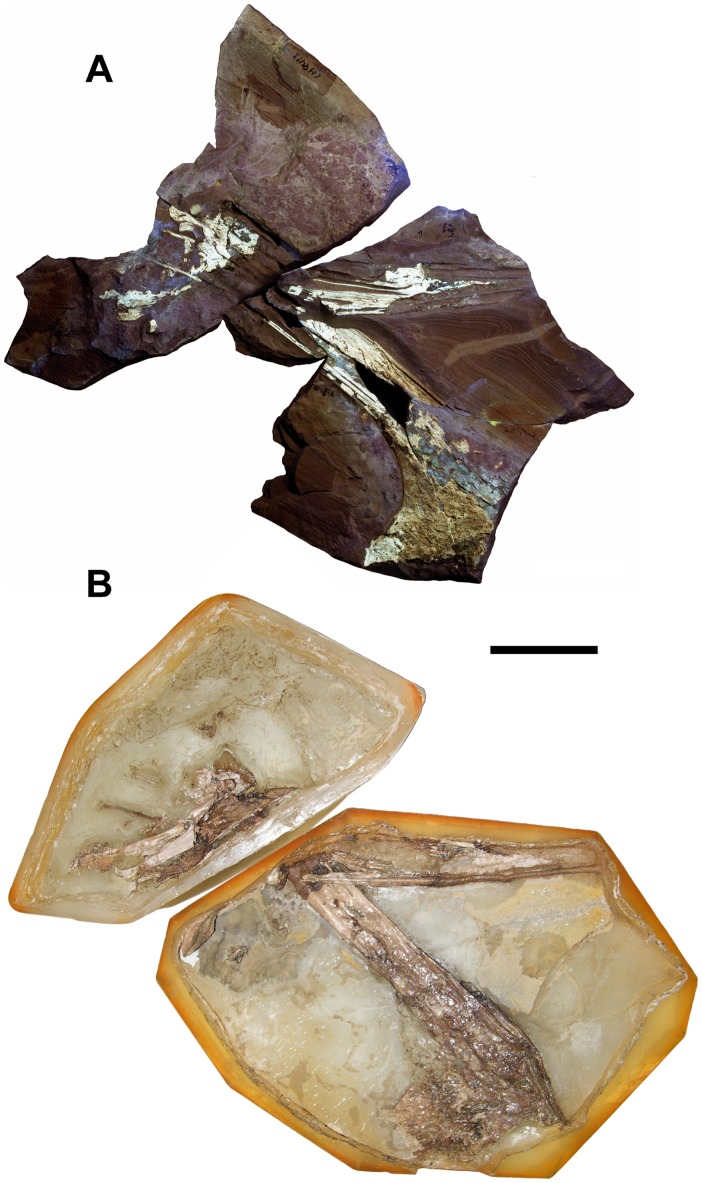
Photographs of the holotype of *Europejara olcadesorum* gen. et sp. nov. (MCCM-LH 9413). (A) Main slab under ultraviolet light. (B) Acid-prepared counterslab. Scale bar: 50 mm.

**Figure 2:**
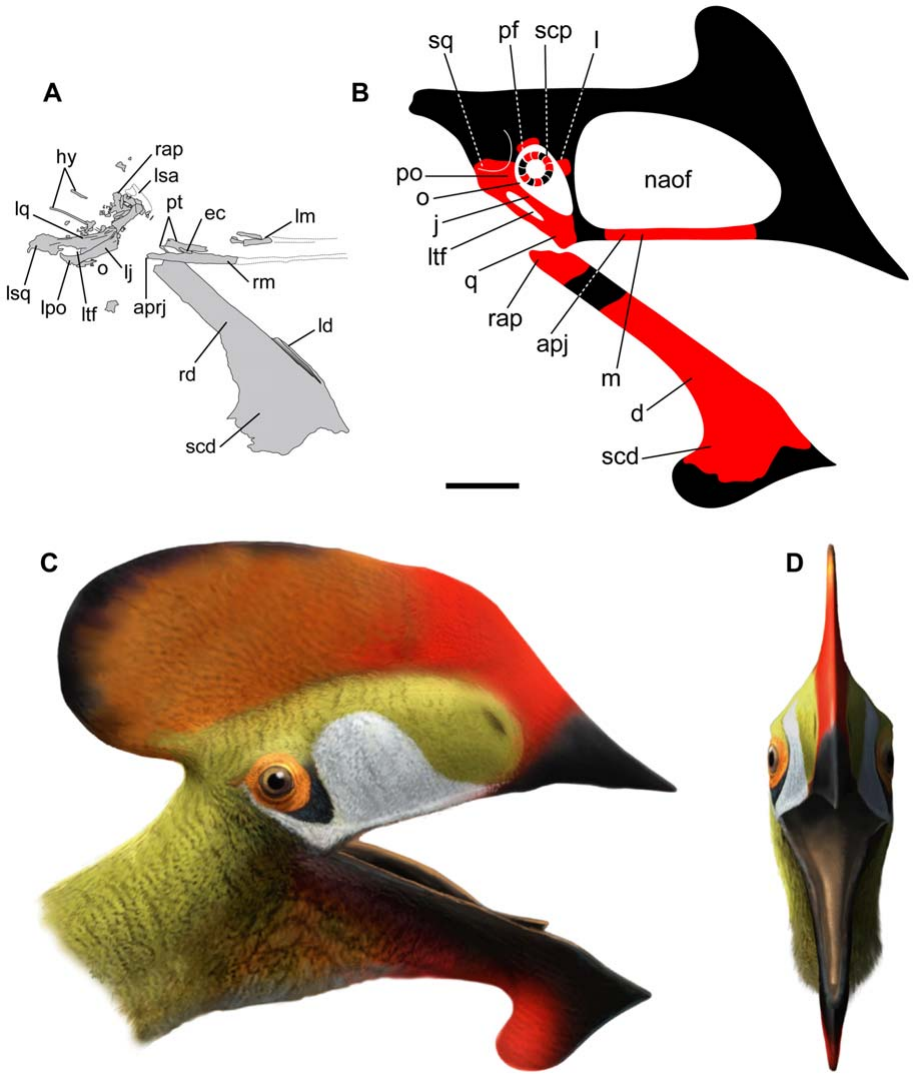
Line drawing of the holotype of *Europejara olcadesorum* gen. et sp. nov. and life restoration. (A) Interpretative line drawing of the skull as observed on the acid-prepared counterslab. (B) Reconstruction of the skull (based in part on *Tapejara*) showing preserved parts in red. Life restoration of the head of *Europejara* in lateral (C) and frontal (D) views. apj, anterior process of the jugal; aprj, anterior process of the right jugal; d, dentary; ec, ectopterygoid; hy, hyoids; j, jugal; l, lacrimal; ld, left dentary; lj, left jugal; lm, left maxilla; lpo, left postorbital; lq, left quadrate; lsa, left surangular; lsq, left squamosal; ltf, lower temporal fenestra; m, maxilla; naof, nasoantorbital fenestra; o, orbit; pf, postfrontal; po, postorbital; pt, pterygoid; q, quadrate; rap, retroarticular process; rd, right dentary; rm, right maxilla; scd, sagittal crest of the dentary; scp, scleral plates; sq, squamosal. Scale bar: 50 mm.

**Figure 3:**
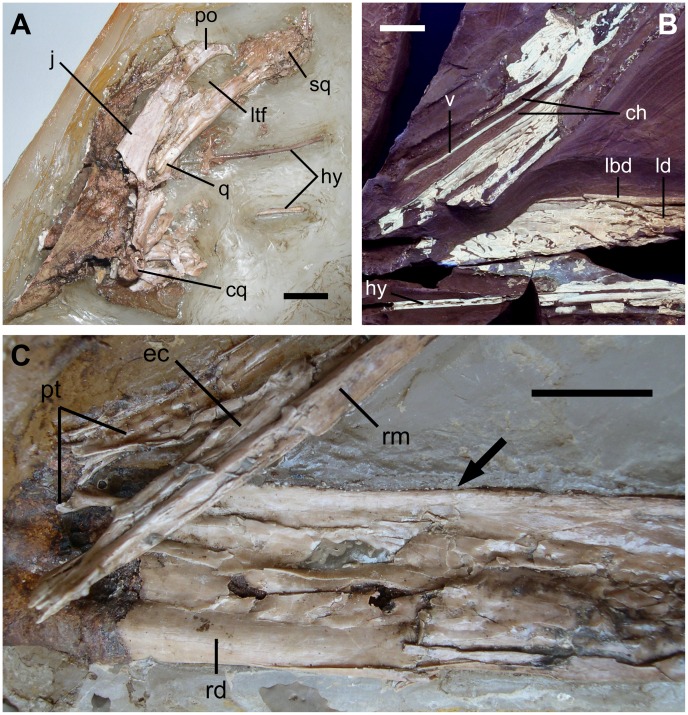
Skull of *Europejara olcadesorum* gen. et sp. nov. (A) Close-up of the crushed left post-orbital region of the skull (acid-prepared counterslab) in lateral view showing the narrowness of the lower temporal fenestra (ltf), the mandibular condyle of the quadrate (cq) and the distal extremities of the hyoid apparatus (hy). (B) Close-up of the posterior area of the palate (main slab under ultraviolet light) in dorsal view showing the thin, elongated vomer (v) septum separating the two choanae (ch). Note the medial surface of the left dentary (ld) and the hyoid apparatus (hy), adjacent to the ventral margin of the mandible. (C) Detail of the posterior area of the palate (acid-prepared counterslab) in dorsal view showing the pterygoid (pt), the ectopterygoid (ec), and the right maxilla (rm). Note the robustness of right mandibular ramus (in lateral view) and its thin, edentulous dorsal edge (arrow). ch, choanae; cq, mandibular condyle of the quadrate; ec, ectopterygoid; hy, hyoid apparatus; j, jugal; lbd, lingual bulge of the dentary; ld, left dentary; lft, lower temporal fenestra; po, postorbital; pt, pterygoid; q, quadrate; rd, right dentary; rm, right maxilla; sq, squamosal; v, vomer. Scale bars: 10 mm.

**Figure 4:**
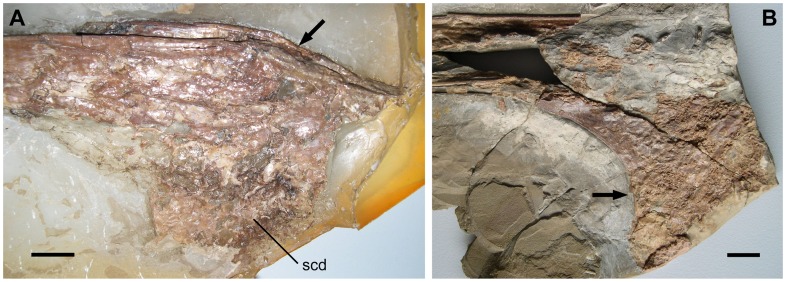
Lower jaw of *Europejara olcadesorum* gen. et sp. nov. (A) Close-up of the symphyseal area (acid-prepared counterslab) in right lateral view showing the typical step-like dorsal margin of the dentary in tapejarines (arrow). Note the strong lateral compression of the mandible and the trabecular structure of the sagittal crest of the dentary (scd). (B) Close-up of the best preserved margin of the dentary crest (main slab). Note the concave posterior border (arrow) giving the peculiar recurved aspect of the dentary crest of *Europejara*. scd, sagittal crest of the dentary. Scale bars: 10 mm.

MCCM-LH 9413, jaws and post-orbital elements of the skull preserved on part and counterpart slabs (Figures 1, 2, 3, 4). Specimen housed in the Museo de las Ciencias de Castilla–La Mancha (MCCM), Cuenca, Spain.

#### Locality and Horizon

Las Hoyas (Cuenca, Spain), upper Barremian laminated limestone of the La Huérguina Formation (Lower Cretaceous) [18], [28].

#### Diagnosis

Tapejarine tapejarid with the following combination of characters that distinguish it from other members of the clade (autapomorphies marked with an asterisk*): dentary bony sagittal crest caudally recurved*; dentary crest more developed dorsoventrally than anteroposteriorly*; high dentary crest height/mandibular ramus height (DCH/MRH) ratio* (found to be about 4 whereas it ranges from 1.5 to 3 in other tapejarid species); presence of a lingual bulge developing medially along the dorsal border of the dentary; presence of some shallow but well marked depressions on the medial surface of the mandibular rami.

### Description and Comparisons

The skull is incomplete, crushed, and flattened dorsoventrally, whereas the mandible is preserved in lateral view (Figure 1). The bones of the left post-orbital region of the skull (jugal postorbital process, postorbital, quadrate, and squamosal) are crushed and displaced. Some small bones, including a fragmentary lacrimal and a possible postfrontal, were preserved around the orbital region and have been removed from the matrix during the acid preparation. No integumentary structures (e.g. soft tissue, keratinous rhamphotheca) are preserved.

Both maxillae are incomplete. They are exposed dorsally and form an angle of about 12°. The posterior end of the right maxilla is apparently still in contact with the anterior process of the jugal, but no suture can be observed. Only the left jugal, missing its anterior and ascending processes, can be unambiguously identified. Its preserved part corresponds to the process that is in contact with the postorbital. This robust process separates the orbit from the lower temporal fenestra. It decreases in width from its base to the contact area with the postorbital. Some longitudinal folds are present at its base. The incomplete element that is interpreted as a fragmentary lacrimal is thin and fenestrated, a typical feature of tapejarine pterosaurs (e.g. [6]).

The postorbital is roughly triangular, and no suture can be observed between this bone and the underlying process of the jugal (Figure 3A). The left quadrate is located posterior to the preserved process of the jugal, thus determining the approximate position of the narrow lower temporal fenestra. The quadrate bears medially a thin bony lamella, as well as a few longitudinal folds. The mandibular articulation of the quadrate shows two rounded condyles. The squamosal appears as a bony mass whose outline is poorly defined. It is in connection with the quadrate.

The palate, exposed in dorsal view, shows two narrow and elongate choanae separated by a thin vomer (Figure 3B). The sutures between the palatine, pterygoid, ectopterygoid and maxilla are poorly visible. A process of the pterygoid–ectopterygoid complex projects anterolaterally and connects to the posterior process of the maxilla. It separates two oval-shaped fenestrae posterolaterally to the choanae (Figure 3C). A shallow depression is present on the anterior surface of this pterygoid–ectopterygoid complex process.

Some trapezoidal-shaped scleral plates are also preserved. Most of these thin bony elements, now removed from the matrix, were originally preserved in the orbital region of the skull. A few other scleral plates are scattered on the slab surface.

**Figure 5:**
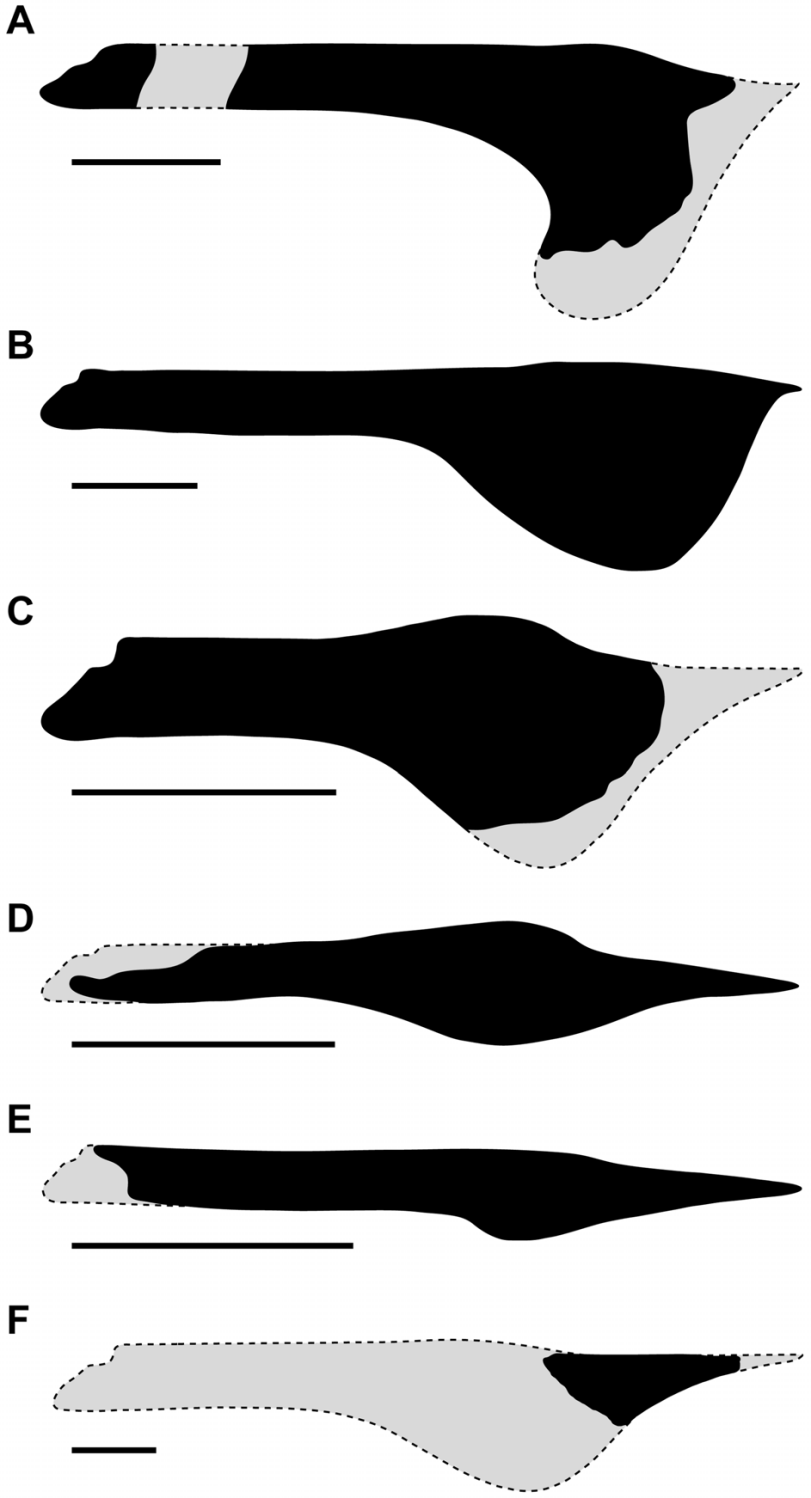
Comparisons of tapejarine lower jaws. (A) *Europejara olcadesorum* gen. et sp. nov. (specimen MCCM-LH 9413): dentary crest height/mandibular ramus height (DCH/MRH) ratio = 4. (B) *Tupandactylus imperator* (specimen CPCA 3590): DCH/MRH ratio = 3. (C) *Tapejara wellnhoferi* (specimen AMNH 24440): DCH/MRH ratio = 2.5. (D) *Sinopterus dongi* (specimen GMN-03-11-001, holotype of *Huaxiapterus jii*): DCH/MRH ratio = 2.2. (E) *Sinopterus dongi* (specimen IVPP V 13363): DCH/MRH ratio = 1.5. (F) Kem Kem tapejarine (specimen BSP 1997 I 67): DCH/MRH ratio unknown. Gray areas indicate missing parts. Scale bars: 50 mm.

The mandible is nearly complete with a preserved length of 230 mm and an estimated total length of 255 mm. Although most of the preserved portion of the mandible corresponds to the dentaries, some posterior elements can be identified as the surangular and the retroarticular process. The mandibular rami (22 mm in height) are robust and show parallel dorsal and ventral borders. The lateral surface of the ramus is smooth whereas the medial surface displays some shallow but well-marked depressions. A lingual bulge is present medially along the dorsal border of the dentaries. In lateral view, the cutting edge of the dentaries is slightly sigmoid at the symphyseal area, being convex posteriorly and concave anteriorly. The dorsal surface of the symphysis is concave in cross-section. The mandible of *Europejara* bears a deep dentary bony sagittal crest that is recurved caudally. The estimated height of this crest is about 90 mm, giving a DCH/MRH ratio of about 4 (Figure 5A).

The hyoid apparatus, represented by the ossified first pair of rod-like ceratobranchials, lies adjacent to the ventral margin of the mandible. The branches are about 135 mm in length and less than 2 mm in diameter, and are slightly curved in their posterior half (Figure 3A).

*Europejara* is mainly distinguished from the Chinese tapejarines by having a larger size and in having a more developed, deeper dentary sagittal crest. In *Sinopterus dongi*, the DCH/MRH ratio is between 1.5 (value based on the specimen IVPP V 13363 [12]) and 2.2 (value based on the specimen GMN-03-11-001, the holotype of *Huaxiapterus jii* [38], itself a junior synonym of *S. dongi* [39]) (Figure 5D, 5E). The dentary sagittal crest is even less developed in both “*Huaxiapterus*” *corollatus* and “*Huaxiapterus*” *benxiensis* (two species still needing a new generic name) than in *Sinopterus dongi* [40], [41]. Finally, the rare Chinese genus *Eopteranodon* has a skull which remains poorly known. Judging from the figures provided by Lü & Zhang [19], the dentary sagittal crest seems to be similar to that of *Sinopterus*.

The mandible of *Europejara* differs from that of *Tapejara* in displaying a dorsal border less sigmoid at the symphyseal area in lateral view and in having less robust and relatively thinner rami. The DCH/MRH ratio is 2.5 in *Tapejara* (value based on the reconstructed mandible of the specimen AMNH 24440 [11]) (Figure 5C). In juvenile individuals in which the dentary crest is less pronounced [42], [43], this ratio tends to be lower (around 2). The dentary crest is more developed anteroposteriorly in *Tapejara*, occupying approximately the anterior half of the mandible.

The mandibles of *Europejara* and *Tupandactylus* are rather similar in size and proportions, with slender mandibular rami and a deep sagittal crest [44]. However, the DCH/MRH ratio is lower in *Tupandactylus*, with a value of 3 (value based on the specimen CPCA 3590 [44]) (Figure 5B). The dentary sagittal crest of *Tupandactylus* is more developed anteroposteriorly and markedly different in shape, with a more abrupt anterior margin than the posterior one. In addition, the dorsal border of the mandible of *Tupandactylus* is only slightly convex at the symphyseal area, and thus not displaying the sigmoid shape observed in *Europejara*.

The condition of the specimen BSP 1997 I 67 from the early Cenomanian Kem Kem beds in Morocco is too fragmentary for a significant comparison with the mandible of *Europejara*. The Kem Kem specimen corresponds only to the anterior portion of the mandibular symphysis [13]. However, the development of a deep dentary sagittal crest [6], [13] would indicate that this specimen is a tapejarine. Based on the mandible of *Tapejara*, a reconstruction suggests that the lower jaw of the Moroccan tapejarine may have been larger (about 450 mm in length), and the tip of the rostrum much more pointed than in *Europejara* (Figure 5F).

Among other azhdarchoids, *Europejara* clearly differs from chaoyangopterids (i.e., *Chaoyangopterus*, *Jidapterus*, *Shenzhoupterus*), thalassodromine tapejarids (i.e., *Thalassodromeus*, *Tupuxuara*), and azhdarchids (i.e., *Azhdarcho*, *Bakonydraco*, *Zhejiangopterus*, *Quetzalcoatlus*) by the presence and the shape of the dentary sagittal crest. The lower jaw of the latter three groups is more elongate (i.e., with a higher length/height ratio) and has a dentary sagittal crest which is very low or apparently absent.

**Figure 6:**
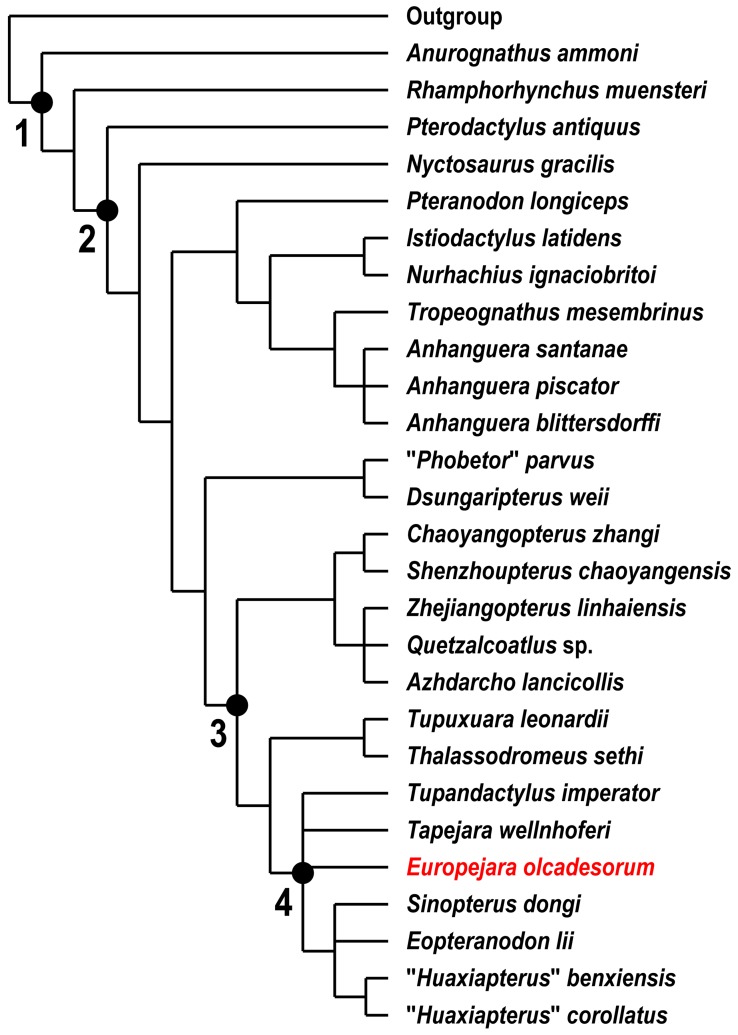
Strict consensus cladogram showing the relationships of *Europejara olcadesorum* gen. et sp. nov. to other Pterodactyloidea. *Europejara olcadesorum* was found to be a member of the clade Tapejarinae (*sensu* [7]). Tree length = 205, consistency index = 0.7561, retention index = 0.8538, rescaled consistency index = 0.6456 (see Appendix S1 for character list and data matrix). Nodes: 1, Pterosauria; 2, Pterodactyloidea; 3, Azhdarchoidea; 4, Tapejarinae (*sensu* [7]). As indicated by its toothless condition, *Europejara* is included within the clade Azhdarchoidea (synapomorphy: character 57, state 3). *Europejara* shares with other tapejarines the apomorphic feature consisting of the presence of a step-like dorsal margin of the dentary in lateral view (synapomorphy: character 52, state 1).

A strict consensus cladogram of the phylogenetic analysis is shown in Figure 6. Our results place *Europejara* as a member of the Tapejaridae. This group is currently divided into two clades: the Thalassodrominae and the Tapejarinae (*sensu* [7]). *Europejara olcadesorum* can clearly be distinguished from the thalassodromines which are large pterosaurs (wingspans around 4 meters) with peculiar large premaxillary crests and straight beaks. The new taxon, on the contrary, is comparatively smaller (estimated wingspan around 2 meters, based on proportions of other tapejarines) and shares derived features present in the Tapejarinae, including the presence of a bony dentary sagittal crest. Furthermore, *Europejara* possesses an unambiguous tapejarine synapomorphy: a peculiar step-like, sigmoid dorsal dentary edge at the symphyseal area. However, its relationships within the clade Tapejarinae (*sensu* [7]) remain poorly resolved (Figure 6). *Europejara* differs from *Sinopterus* (regarded as the senior synonym of *Huaxiapterus*; see [39]) from the Jiufotang Formation [12], [38] and *Tapejara* from the Santana Formation [5], [11] in having a visibly deeper dentary bony sagittal crest that is recurved caudally. The length of the palate and mandible indicates that the skull of *Europejara* was relatively more elongate than that of *Tapejara*, but shorter than that of *Tupandactylus* from the Crato Formation [7], [11], [44].

The mandibular crest of *Europejara* displays a unique shape never before observed in previously described pterosaurs. The purpose of such a deep and recurved dentary sagittal crest remains unclear but it may have served multiple functions (e.g. aerodynamics, thermoregulation, social behavior, and/or gular pouch supportive structure). While well-developed cranial bony crests have been reported for various groups of pterosaurs, mandibular crests are only known in ornithocheirids ( =  anhanguerids) and tapejarids [15], [45], in addition to some primitive non-pterodactyloid pterosaurs [46]. Among Tapejaridae, both premaxillary and dentary crests are low in *Sinopterus* and “*Huaxiapterus*”, whereas they are well-developed in *Tapejara* and *Tupandactylus* [44]. It can thus be inferred that the dimensions of both crests were roughly correlated in tapejarids. Accordingly, the deep dentary crest of *Europejara* suggests the presence of a relatively high premaxillary sagittal crest (Figure 2 B–D), as in *Tapejara* and *Tupandactylus*. Such a premaxillary sagittal crest might also have been caudally recurved, mimicking the condition of the dentary crest.

## Discussion

**Figure 7:**
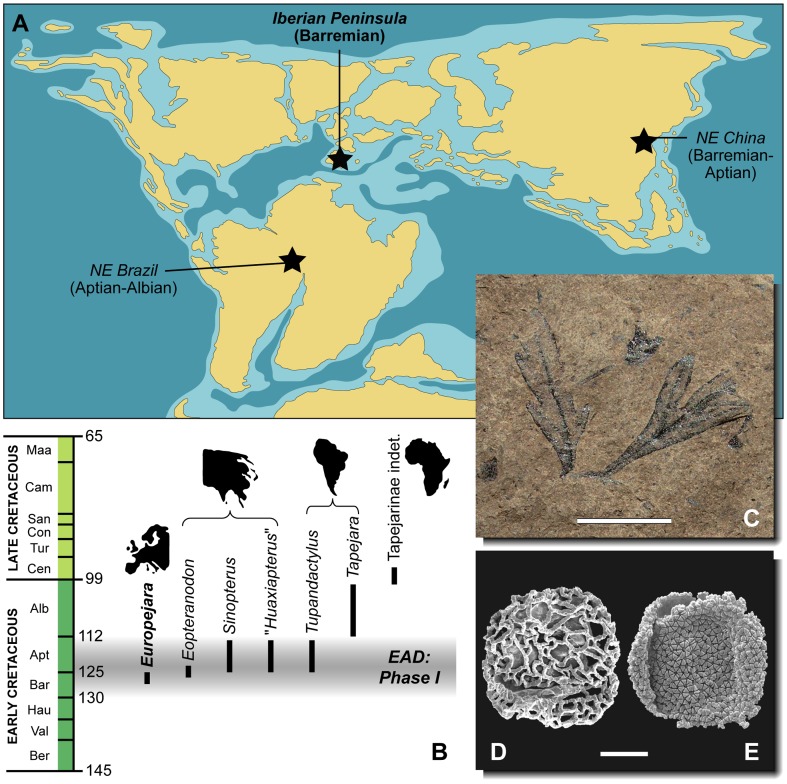
Distribution through time and space of tapejarine pterosaurs and early angiosperms. (A) Early Cretaceous (Aptian) paleogeographical map showing the three main areas where tapejarids co-occur with early angiosperms (black stars). (B) Stratigraphic distribution of the different tapejarid taxa (silhouettes above each taxon denote geographical occurrences); note that the diversification of tapejarids coincides with the Phase I of the early angiosperm diversification (EAD) [60]–[62]. (C) Leaves of an early angiosperm (cf. *Jixia*) from the late Barremian of Las Hoyas (MCCM-LH 30351), one of the oldest known macrofossil of a terrestrial flowering plant. Early angiosperm pollen grains *Afropollis* (D) and *Stellatopollis* (E) from the La Huérguina Formation, both worldwide distributed during the Barremian–Aptian interval. Ber, Berriasian; Val, Valanginian; Hau, Hauterivian; Bar, Barremian; Apt, Aptian; Alb, Albian; Cen, Cenomanian; Tur, Turonian; Con, Coniacian; San, Santonian; Cam, Campanian; Maa, Maastrichtian. Scale bars: 10 mm (C) and 10 µm (D,E).

The presence of a tapejarid in the Barremian of Spain stresses that the distribution of this group was scattered throughout the Gondwanan and Laurasian landmasses bounded by the Tethyan Ocean, from Brazil to the Iberian Peninsula and China (Figure 7). Although *Europejara* is definitively a tapejarid, the information provided by the fossil to resolve its phylogenetic affinities is partially incomplete. Notwithstanding this, the phylogenetic result does not reject the hypothesis of *Europejara* being the sister group of a clade grouping the Brazilian and Chinese taxa. In fact, this would be the most stratigraphically congruent result; proposing any of the Brazilian taxa as the closest relatives of clade grouping *Europejara* plus the Chinese tapejarines would be inconsistent unless older taxa were found in the Gondwanan masses. Furthermore, the fact that the earliest known members of the Tapejaridae correspond to the forms found in the late Barremian of the La Huérguina Formation (this study) and around the Barremian–Aptian boundary of the Yixian Formation [9], [10] reinforces the view that the group may well have originated in Eurasia, rather than in Gondwana [9]. Later, in the Aptian, tapejarids may have extended their geographic distribution, reaching the northeastern parts of Brazil. Lastly, the occurrence of a tapejarid in the Cenomanian of North Africa (Morocco) [6], [13], [14] suggests a possible Gondwanan diversification during the mid-Cretaceous. However, the knowledge of the evolutionary and paleobiogeographic history of Tapejaridae is affected by an uneven fossil record sampling [2], [47]. Indeed, their apparent absence in the Aptian–Albian deposits of North America, Europe and Africa may be due to the lack of pterosaur-bearing Konservat-Lagerstätten of this age in those continents.

During the evolutionary history of Pterosauria, total edentulism (i.e. the complete loss of teeth) occurred twice [24] or most likely at least three times [20], [25], [26]. All members of Azhdarchoidea reported to date and known by cranial and mandibular material, ranging from the Aptian to the Maastrichtian, were toothless forms. In addition, some edentulous lineages evolved among non-azhdarchoid pterodactyloids (e.g. *Pteranodon, Geostenbergia, Nyctosaurus*) during the Late Cretaceous [24], [25], [39]. Thus, the Barremian tapejarid *Europejara olcadesorum* represents the oldest known unambiguous toothless pterosaur as directly evidenced by the edentulous jaws preserved in MCCM-LH 9413. An isolated humerus from the Barremian part of the Wessex Formation in southern England was assigned to an indeterminate non-azhdarchid azhdarchoid pterosaur [48], and therefore may also correspond to a toothless form. Recently, the genus *Aurorazhdarcho* from the Late Jurassic Solnhofen Limestone (Tithonian) of southern Germany was described as the oldest known azhdarchoid, but the only known specimen is a postcranial skeleton [49].

Tapejarines had well-developed flying skills and an excellent visual system [42], and have mostly been regarded as frugivorous or seed-eating forms on the basis of the shape and of their edentulous jaws [4], [9], [11], [12]. Furthermore, there is evidence that their beak may have been covered rostrally by a rhamphotheca [8], [44]. Although the rhamphotheca would have been basically shaped by the underlying bones, like in modern birds, the cutting edges of the keratinous bill of some tapejarines might have borne one or more pointed projections as in the omnivorous rhamphastid birds (i.e. toucans) [50], [51]. Despite the fact that this analogy is not supported by direct fossil evidence, this suggests that, if not fully herbivorous, an omnivorous diet including seeds and/or fruits, insects and small vertebrates can be addressed for tapejarines.

The concept of a Cretaceous Terrestrial Revolution (KTR, a period of major reorganization of ecosystems in the Cretaceous, as termed by Lloyd et al. [27]) associates the diversification of angiosperms with that of insects, birds and mammals, but does not integrate pterosaurs within this macroevolutionary event. As far as the vertebrate–angiosperm interactions are concerned, an efficient and successful dispersal mode would have been a key mechanism at the origin of the rapid Early Cretaceous cosmopolitanism of angiosperms. In this sense, the internal vertebrate dispersal mode [52], [53] would have favored the colonization by flowering plants of new, distant areas.

The fossil assemblages of the Early Cretaceous localities of the La Huérguina Formation in Spain and the Yixian and Jiufotang formations in northeastern China contain organisms which may have inhabited mosaic of subtropical wet, forested and lacustrine environments [28], [31], [54], [55]. The presence and abundance of early angiosperms in these regions [56]–[58] would support the hypothesis that frugivorous–granivorous pterosaurs have existed in such paleoecosystems and thus could have been angiosperm seed dispersers [59], together with insects and birds. Tapejarines not only were members of the trophic networks throughout the KTR, but their morphological innovations, in turn, may have been tied to the dispersal of early angiosperms. In fact, tapejarines and early flowering plants display synchronic radiation events and similar patchy geographic distributions that appear to covary (i.e. temporal and spatial congruence). In light of this, the Barremian–Aptian distribution of tapejarines might be partially associated with the first radiation phase of the early angiosperm plants occurring at that time in both hemispheres [60]–[62] (Figure 7A, 7B).

This first phase of the early angiosperm diversification is particularly well documented in the Iberian Peninsula with about 50 taxa recently being recognized from the late Barremian–early Aptian of Portugal [63]. In the late Aptian–early Albian mesofossil flora of the Portuguese Famalicão locality, about 110 species of angiosperms have been identified, with a high proportion of fleshy-fruited forms [53], [63]. Dispersal systems of such species have been interpreted as endozoochorous (i.e. berries and drupes). Palynomorph assemblages from the La Huérguina Formation show a varied spectrum of early angiosperm pollen grains (Figure 7E, 7F). Although most terrestrial plant microfossils collected in this formation belong to pteridophytes and gymnosperms, there is a significant amount of angiosperm pollen grains *(Afropollis, Clavatipollenites, Retimonocolpites, Stellatopollis, Transitoripollis*). In addition, angiosperm leaf macrofossils are relatively common at Las Hoyas [64] (Figure 7C). Moreover, the Aptian–Albian Crato and Santana formations from which the Brazilian tapejarines have been recovered are also famous for their rich and diverse early angiosperm assemblages [65], [66]. Thus, all this evidence provides a spatial and temporal congruence between the early radiation of angiosperms and tapejarines, although further analyses are necessary to address if this association involved the co-evolution of these groups (see [67]), or whether tapejarines were more likely incidental vectors.

In conclusion, *Europejara* is the first tapejarid pterosaur described from Europe and represents the oldest known edentulous pterosaur. This discovery documents an important stage in the evolutionary history of pterodactyloids, in which the great cranial morphological disparity observed during the Early Cretaceous may reflect a broadening of feeding habits. From a paleoecological point of view, it can reasonably be assumed that herbivorous pterosaurs (most likely including tapejarines) existed and were one of the biological vectors involved in the dispersal of early angiosperms between the different landmasses of the Early Cretaceous world. However, this hypothesis will need to be tested and confirmed when more quantitative (i.e. further occurrences) and qualitative (i.e. gut contents) data are available.

## Supporting Information

Appendix S1 Phylogenetic Analysis: Character List, Data Matrix.
(DOC)

## References

[1] Wang X, Kellner AWA, Zhou Z, Campos DA (2005). Pterosaur diversity and faunal turnover in Cretaceous terrestrial ecosystems in China.. Nature.

[2] Butler RJ, Barrett PM, Nowbath S, Upchurch P (2009). Estimating the effects of sampling biases on pterosaur diversity patterns: implications for hypotheses of bird/pterosaur competitive replacement.. Paleobiology.

[3] Dyke GJ, McGowan AJ, Nudds RL, Smith D (2009). The shape of pterosaur evolution: evidence from the fossil record.. J Evol Biol.

[4] Prentice KC, Ruta M, Benton MJ (2011). Evolution of morphological disparity in pterosaurian.. J Syst Palaeontol.

[5] Kellner AWA (1989). A new edentate pterosaur of the Lower Cretaceous from the Araripe Basin, Northeast Brazil.. An Acad bras Cienc.

[6] Kellner AWA (2004). New information on the Tapejaridae (Pterosauria, Pterodactyloidea) and discussion of the relationships of this clade.. Ameghiniana.

[7] Kellner AWA, Campos D (2007). Short note on the ingroup relationships of the Tapejaridae (Pterosauria, Pterodactyloidea).. Bol Mus Nac N S Geol.

[8] Kellner AWA, Campos DA (2002). The function of the cranial crest and jaws of a unique pterosaur from the Early Cretaceous of Brazil.. Science.

[9] Wang X, Zhou Z (2006). Pterosaur assemblages of the Jehol Biota and their implication for the Early Cretaceous pterosaur radiation.. Geol J.

[10] Kellner AWA, Wang X, Zhou Z, Campos D (2007). On a new tapejarid (Pterosauria, Pterodactyloidea) from the Cretaceous Yixian Formation (Jehol Biota, China): the oldest toothless pterosaur.. J Vert Paleontol 27 (suppl to.

[11] Wellnhofer P, Kellner AWA (1991). The skull of *Tapejara wellnhoferi* Kellner (Reptilia, Pterosauria) from the Lower Cretaceous Santana Formation of the Araripe Basin, Northeastern Brazil.. Mitt Bayer Staatsslg Paläont hist Geol.

[12] Wang X, Zhou Z (2003). A new pterosaur (Pterodactyloidea, Tapejaridae) from the Early Cretaceous Jiufotang Formation of western Liaoning, China and its implications for biostratigraphy.. Chin Sci Bull.

[13] Wellnhofer P, Buffetaut E (1999). Pterosaur remains from the Cretaceous of Morocco.. Paläontol Z.

[14] Ibrahim N, Unwin DM, Martill DM, Baidder L, Zouhri S (2010). A new pterosaur (Pterodactyloidea: Azhdarchidae) from the Upper Cretaceous of Morocco.. PLoS ONE.

[15] Wellnhofer P (1991). The Illustrated Encyclopedia of Pterosaurs. London: Salamander Books.. 192 p.

[16] Unwin DM (2001). An overview of the pterosaur assemblage from the Cambridge Greensand (Cretaceous) of Eastern England.. Mitt Mus Nat.kd Berl Geowiss Reihe.

[17] Barrett PM, Butler RJ, Edwards NP, Milner AR (2008). Pterosaur distribution in time and space: an atlas.. Zitteliana.

[18] Sanz JL, Fregenal-Martínez MA, Meléndez N, Ortega F, Briggs DEG, Crowther PR (2001). Las Hoyas..

[19] Lü JC, Zhang BK (2005). New pterodactyloid pterosaur from the Yixian Formation of Western Liaoning.. Geol Rev.

[20] Andres B, Ji Q (2008). A new pterosaur from the Liaoning Province of China, the phylogeny of the Pterodactyloidea, and the convergence in their cervical vertebrae.. Palaeontology.

[21] Chang S, Zhang H, Renne PR, Fang Y (2009). High-precision ^40^Ar/^39^Ar age for the Jehol Biota.. Palaeogeogr Palaeoclimatol Palaeoecol.

[22] Zanno LE, Makovicky PJ (2011). Herbivorous ecomorphology and specialization patterns in theropod dinosaur evolution.. Proc Natl Acad Sci USA.

[23] Zheng X, Martin LD, Zhou Z, Burnham DA, Zhang F (2011). Fossil evidence of avian crops from the Early Cretaceous of China.. Proc Natl Acad Sci USA.

[24] Unwin DM, Buffetaut E, Mazin J-M (2003). On the phylogeny and evolutionary history of pterosaurs.. Geological Society, London, Special Publications.

[25] Kellner AWA, Buffetaut E, Mazin J-M (2003). Pterosaur phylogeny and comments on the evolutionary history of the group.. Geological Society, London, Special Publications.

[26] Wang X, Kellner AWA, Jiang S, Meng X (2009). An unusual long-tailed pterosaur with elongated neck from western Liaoning of China.. An Acad Bras Cienc.

[27] Lloyd GT, Davis KE, Pisani D, Tarver JE, Ruta M (2008). Dinosaurs and the Mesozoic Terrestrial Revolution.. Proc R Soc B.

[28] Fregenal-Martínez MA, Meléndez N, Gierlowski-Kordesch EH, Kelts KR (2000). The lacustrine fossiliferous deposits of the Las Hoyas sub-basin (Lower Cretaceous, Serranía de Cuenca, Iberian Ranges, Spain).. AAPG Studies in Geology.

[29] Buscalioni AD, Fregenal MA, Bravo A, Poyato-Ariza FJ, Sanchíz B (2008). The vertebrate assemblage of Buenache de la Sierra (Upper Barremian of Serrania de Cuenca, Spain) with insights into its taphonomy and palaeoecology.. Cret Res.

[30] Sanz JL, Wenz S, Yébenes A, Estes R, Martínez-Delclòs X (1988). An Early Cretaceous faunal and floral continental assemblage: Las Hoyas fossil site (Cuenca, Spain).. Geobios.

[31] Buscalioni AD, Fregenal-Martínez MA (2010). A holistic approach to the palaeoecology of Las Hoyas Konservat-Lagerstätte (La Huérguina Formation, Lower Cretaceous, Iberian Ranges, Spain).. J Iber Geol.

[32] Ortega F, Escaso F, Sanz JL (2010). A bizarre, humped Carcharodontosauria (Theropoda) from the Lower Cretaceous of Spain.. Nature.

[33] Buscalioni AD, Fregenal-Martínez MA, Barrett PM, Evans SE (2006). Archosaurian size bias in Jurassic and Cretaceous freshwater ecosystems..

[34] Vullo R, Buscalioni AD, Marugán-Lobón J, Moratalla JJ (2009). First pterosaur remains from the Early Cretaceous Lagerstätte of Las Hoyas, Spain: palaeoecological significance.. Geol Mag.

[35] Briggs DEG, Wilby PR, Pérez-Moreno BP, Sanz JL, Fregenal-Martínez MA (1997). The mineralization of dinosaur soft-tissue in the Lower Cretaceous of Las Hoyas.. J Geol Soc.

[36] Swofford DL (2000). PAUP*. Phylogenetic Analysis Using Parsimony (*and other methods). Version 4.. Sunderland: Sinauer Associates.

[37] Wang X, Kellner AWA, Jiang S, Cheng X (2012). New toothed flying reptile from Asia: close similarities between early Cretaceous pterosaur faunas from China and Brazil.. Naturwissenschaften.

[38] Lü JC, Yuan CX (2005). New tapejarid pterosaur from western Liaoning, China.. Acta Geol Sin.

[39] Kellner AWA (2010). Comments on the Pteranodontidae (Pterosauria, Pterodactyloidea) with the description of two new species.. An Acad Bras Cienc.

[40] Lü J, Jin X, Unwin DM, Zhao L, Azuma Y (2006). A new species of *Huaxiapterus* (Pterosauria: Pterodactyloidea) from the Lower Cretaceous of western Liaoning, China with comments on the systematics of tapejarid pterosaurs.. Acta Geol Sin.

[41] Lü J, Gao Y, Xing L, Li Z, Ji Q (2007). A new species of *Huaxiapterus* (Pterosauria: Tapejaridae) from the Early Cretaceous of western Liaoning, China.. Acta Geol Sin.

[42] Eck K, Elgin RA, Frey E (2011). On the osteology of *Tapejara wellnhoferi* Kellner 1989 and the first occurrence of a multiple specimen assemblage from the Santana Formation, Araripe Basin, NE-Brazil.. Swiss J Palaeontol.

[43] Elgin RA, Campos HBN (2012). A new specimen of the azhdarchoid pterosaur *Tapejara wellnhoferi*.. Hist Biol 24: In press.

[44] Pinheiro FL, Fortier DC, Schultz CL, Andrade JAFG de, Bantim RAM (2011). New information on the pterosaur *Tupandactylus imperator*, with comments on the relationships of Tapejaridae.. Acta Palaeontol Pol.

[45] Frey E, Martill DM, Buchy M-C, Buffetaut E, Mazin J-M (2003). A new species of tapejarid pterosaur with soft-tissue head crest.. Geological Society, London, Special Publications.

[46] Stecher R (2008). A new Triassic pterosaur from Switzerland (Central Austroalpine, Grisons), *Raeticodactylus filisurensis* gen. et sp. nov.. Swiss J Geosci.

[47] Butler RJ, Brusatte SL, Andres B, Benson RBJ (2012). How do geological sampling biases affect studies of morphological evolution in deep time? A case study of pterosaur (Reptilia: Archosauria) disparity.. Evolution.

[48] Witton MP, Martill DM, Green M (2009). On pterodactyloid diversity in the British Wealden (Lower Cretaceous) and a reappresial of “*Palaeornis*” *cliftii* Mantell, 1844.. Cret Res.

[49] Frey E, Meyer CA, Tischlinger H (2011). The oldest azhdarchoid pterosaur from the Late Jurassic Solnhofen Limestone (Early Tithonian) of Southern Germany.. Swiss J Geosci.

[50] Stettenheim PR (2000). The integumentary morphology of modern birds–An overview.. Amer Zool.

[51] Ősi A, Buffetaut E, Prondvai E (2011). New pterosaurian remains from the Late Cretaceous (Santonian) of Hungary (Iharkút, Csehbánya Formation).. Cret Res.

[52] Webb CJ (1998). The selection of pollen and seed dispersal in plants.. Plant Species Biol.

[53] Eriksson O, Friis EM, Pedersen KR, Crane PR (2000). Seed size and dispersal systems of Early Cretaceous angiosperms from Famalicão, Portugal.. Int J Plant Sci.

[54] Gomez B, Martín-Closas C, Méon H, Thévenard F, Barale G (2001). Plant taphonomy and palaeoecology in the lacustrine Uña delta (Late Barremian, Iberian Ranges, Spain).. Palaeogeogr Palaeoclimatol Palaeoecol.

[55] Zhou Z, Barrett PM, Hilton J (2003). An exceptionally preserved Lower Cretaceous ecosystem.. Nature.

[56] Sun G, Dilcher DL (2002). Early angiosperms from the Lower Cretaceous of Jixi, eastern Heilongjiang, China.. Rev Palaeobot Palynol.

[57] Sun G, Dilcher DL, Zheng S-L (2008). A review of recent advances in the study of early angiosperms from northeastern China.. Palaeoworld.

[58] Coiffard C, Gomez B, Thévenard F (2007). Early Cretaceous angiosperm invasion of Western Europe and major environmental changes.. Ann Bot.

[59] Fleming H, Lips KR (1991). Were pterosaurs Cretaceous seed dispersers?. Am Nat.

[60] Crane PR, Friis EM, Pedersen KR (1995). The origin and early diversification of angiosperms.. Nature.

[61] Wing SL, Boucher LD (1998). Ecological aspects of the Cretaceous flowering plant radiation.. Annu Rev Earth Planet Sci.

[62] Archangelsky S, Barreda V, Passalia MG, Gandolfo M, Prámparo M (2009). Early angiosperm diversification: evidence from southern South America.. Cret Res.

[63] Friis EM, Pedersen KR, Crane PR (2010). Cretaceous diversification of angiosperms in the western part of the Iberian Peninsula.. Rev Palaeobot Palynol.

[64] Barral Cuesta A, Gomez B, Buscalioni AD, Fregenal-Martínez MA (2009). Dicot-like angiosperm leaves from the upper Barremian of Las Hoyas (Serranía de Cuenca, Spain): morphological description and morphometrical approach..

[65] Mohr BAR, Friis EM (2000). Early angiosperms from the Early Cretaceous Crato Formation (Brazil), a preliminary report.. Int J Plant Sci.

[66] Heimhofer U, Hochuli P-A (2010). Early Cretaceous angiosperm pollen from a low-latitude succession (Araripe Basin, NE Brazil).. Rev Palaeobot Palynol.

[67] Butler RJ, Barrett PM, Kenrick P, Penn MG (2009). Diversity patterns amongst herbivorous dinosaurs and plants during the Cretaceous: implications for hypotheses of dinosaur/angiosperm co-evolution.. J Evol Biol.

