# Supplementary material for: A New Crested Pterosaur from the Early Cretaceous of Spain: The First European Tapejarid (Pterodactyloidea: Azhdarchoidea)
Source: PLoS One. 2012 Jul 3;7(7):e38900. doi: 10.1371/journal.pone.0038900 (PMC3389002; doi:10.1371/journal.pone.0038900)
Supplement: Appendix S1 — Phylogenetic Analysis: Character List, Data Matrix. (DOC) [file pone.0038900.s001.doc]

# Supporting Information for:

## A New Crested Pterosaur from the Early Cretaceous of Spain : The First European Tapejarid (Pterodactyloidea: Azhdarchoidea)

Romain Vullo, Jesús Marugán-Lobón, Alexander W. A. Kellner, Angela D. Buscalioni, Bernard Gomez, Montserrat de la Fuente, José J. Moratalla

### Phylogenetic Analysis: Character List, Data Matrix

In order to access the phylogenetic position of *Europejara olcadesorum* gen. et sp. nov., we performed a phylogenetic analysis using PAUP 4.0b10 for Microsoft Windows (Swofford, 2000) using the TBR heuristic searches performed using maximum parsimony (see Appendix S1 for character list and data matrix). Characters were given equal weight and treated unordered (ACCTRAN setting). This analysis is based on previous studies (e.g., Kellner 2003; Unwin 2003; Andres & Ji 2008; Wang et al. 2005, 2009, 2012). The search conducted by PAUP, with *Ornithosuchus longidens* and *Herrerasaurus ischigualastensis* as outgroups, produced 135 equally parsimonious trees (205 steps; consistency index = 0.7561; retention index = 0.8538; rescaled consistency index = 0.6456), from which a strict consensus cladogram was obtained.

CHARACTER LIST (per anatomical region)

SKULL

1. Dorsal margin of the skull (Kellner 2003):

0 - straight or curved downward

1 - concave

2 - only rostrum curved upward

2. Upper and lower jaw (Kellner 2003):

0 - laterally compressed

1 - comparatively broad

3. Rostral part of the skull anterior to the external nares (modified from Kellner 2003):

0 - reduced

1 - elongated (less than half of skull length)

2 - extremely elongated (more than half of skull length)

4. Rostral end of premaxillae/maxillae downturned (Kellner 2003):

0 - absent

1 - present

5. Position of the external naris (Kellner 2003):

0 - above the premaxillary tooth row

1 - displaced posterior to the premaxillary tooth row

6. Process separating the external nares (Kellner 2003):

0 - broad

1 - narrow

7. External naris and antorbital fenestra (Unwin 1995):

0 - separated

1 - confluent forming a nasoantorbital fenestra

8. Naris and antorbital fenestra (modified from Kellner 2003):

0 - shorter than 40% of the skull length

1 - longer than 40% of the skull length

9. Posterior margin of antorbital (or nasoantorbital) fenestra (Unwin 2003):

0 - straight

1 - concave

10. Nasoantorbital (or antorbital) fenestra extending dorsal to the orbit (Wang et al. 2012):

0 - absent

1 - present

11. Shape of the orbit (Andres & Ji 2008):

0 - subcircular

1 - circular

2 - circular, with open ventral margin

3 - piriform (elongated)

12. Orbit comparatively small and positioned very high in the skull (Kellner 2003):

0 - absent

1 - present

13. Position of the orbit relative to the nasoantorbital fenestra (naris + antorbital fenestra) (Kellner 2003):

0 - same level or higher

1 - orbit lower than the dorsal rim of the nasoantorbital fenestra

14. Suborbital opening (Kellner 2003):

0 - absent

1 - present

15. Lower temporal fenestra (Wang et al. 2012):

0 - piriform, with ventral portion wider than dorsal

1 - piriform, with dorsal portion wider than ventral

2 - reduced (slit-like)

16. Premaxillary sagittal crest (Bennett 1994):

0 - absent

1 - present

17. Premaxillary sagittal crest, position (Kellner 2003):

0 - confined to the anterior portion of the skull

1 - starting anterior to the anterior margin of the nasoantorbital fenestra, extending beyond occipital region

2 - starting at about the anterior margin of the nasoantorbital fenestra, reaching the skull roof above the orbit but not extending over the occipital region

3 - starting close or at the anterior portion of the skull and extended over the occipital region

4 - starting at the posterior half of the nasoantorbital fenestra

5 - starting at the middle portion of the nasoantorbital fenestra, extending above the occipital region

18. Premaxillary sagittal crest shape (Andres & Ji 2008):

0 - striated, low with a nearly straight dorsal margin

1 - striated, high, spike-like

2 - round dorsal margin, blade-shaped

3 - smooth, expanded anteriorly and forming a low rod-like extension posteriorly

4 - smooth, starting low anteriorly and very expanded posteriorly

19. Expansion on the anterior part of the premaxillary sagittal crest (Wang et al. 2012):

0 - absent

1 - present

20. Elongated dorsal premaxillary spike-like extension (Martill & Naish 2006):

0 - absent

1 - present

21. Expansion of the premaxillary tip (Kellner 2003):

0 - absent

1 - present, with premaxillary end high

2 - present, with premaxillary end dorsoventrally flattened

22. Posterior ventral expansion of the maxilla (Kellner 2003):

0 - absent

1 - present

23. Maxilla-nasal contact (Unwin 2003):

0 - broad

1 - absent

24. Free nasal process (modified after Kellner 2003):

0 - absent

1 - present

25. Free nasal process position (modified after Kellner 2003):

0 - placed laterally

1 - placed medially

26. Free nasal process size (modified after Kellner 2003):

0 - long, almost reaching the ventral margin of the skull

1 - short

2 - knob-like (extremely reduced)

27. Free nasal process orientation (Wang et al. 2012):

0 - directed anteroventrally

1 - directed ventrally

28. Foramen on nasal process (Kellner 2003):

0 - absent

1 - present

29. Lacrimal extensively fenestrated (Kellner 2004):

0 - absent

1 - present

30. Lacrimal process of the jugal thickness (Kellner 2004):

0 - broad

1 - thin

31. Lacrimal process of the jugal inclination (Wang et al. 2005):

0 - inclined anteriorly

1 - subvertical

2 - inclined posteriorly

32. Pronounced ridge on the lateral side of the jugal (Wang et al. 2012):

0 - absent

1 - present

33. Anterior portion of the frontal rugose (Wang et al. 2005):

0 - absent

1 - present

34. Bony frontal crest (Bennett 1994):

0 - absent

1 - present

35. Bony frontal crest, position (Wang et al. 2012):

0 - confined to the posterior end of the skull

1 - starting above the orbit

2 - starting on the posterior half of the nasoantorbital fenestra

36. Bony frontal crest, shape (Kellner 2003):

0 - reduced and blunt

1 - short and spike-like, dorsally deflected

2 - narrow, directed posteriorly

3 - very high and broad, at least doubling the height of the skull above the orbit, directed posteriorly

4 - high, broad base and fan-shaped

37. Bony parietal crest (Bennett 1994):

0 - absent

1 - present

38. Bony parietal crest shape (Kellner 2003):

0 - blunt

1 - constituting the base of the posterior portion of the cranial crest

39. Posterior region of the skull rounded with the squamosal displaced ventrally (Kellner 2003):

0 - absent

1 - present

40. Position of the quadrate relative to the ventral margin of the skull (Kellner 2003):

0 - vertical or subvertical

1 - inclined about 120° backwards

2 - inclined about 150° backwards

41. Position of the articulation between skull and mandible (Kellner 2003):

0 - under the posterior half of the orbit or further backwards

1 - under the middle part of the orbit

2 - under the anterior half of the orbit or further anterior

42. Helical jaw joint (Bennett 1994):

0 - absent

1 - present

43. Supraoccipital (Kellner 2003):

0 - does not extend backwards

1 - extends backwards

44. Foramen pneumaticum piercing the supraoccipital (Kellner 2003):

0 - absent

1 - present

45. Expanded distal ends of the paroccipital processes (Kellner 2003):

0 - absent

1 - present

46. Palatal ridge (Kellner 2003):

0 - absent

1 - discrete, tapering anteriorly

2 - strong, tapering anteriorly

3 - strong, confined to the posterior portion of the palate

47. Slight expansion of the palate close to the anterior opening of the nasoantorbital (or naris + antorbital) fenestra (Wang et al. 2012):

0 - absent

1 - present

48. Maxilla excluded from the internal naris (Kellner 2003):

0 - absent

1 - present

49. Opening between pterygoids and basisphenoid (interpterygoid opening) (Kellner 2003):

0 - absent or very reduced

1 - present and larger than subtemporal fenestra

2 - present but smaller than subtemporal fenestra

50. Basisphenoid (Kellner 2003):

0 - short

1 - elongated

51. Mandibular symphysis (Kellner 2003):

0 - absent or very short

1 - present, at least 30% of mandible length

52. Step-like dorsal margin of the dentary in lateral view (Wang et al. 2012):

0 - absent

1 - present

53. Anterior tip of the dentary downturned (Kellner 2003):

0 - absent

1 - present

54. Dentary bony sagittal crest (Kellner 2003):

0 - absent

1 - present

55. Dentary bony sagittal crest, position (modified after Kellner 2003):

0 - confined to the anterior third of the lower jaw

1 - extending close to the middle portion of the jaw

56. Dentary bony sagittal crest shape (Wang et al. 2012):

0 - small projection

1 - blade-like

2 - elongated ridge-like

3 - deep, broad in lateral view

4 - shallow

57. Position and presence of teeth (modified after Bennett 1994, Kellner 2003 and Unwin 2003):

0 - teeth present, evenly distributed along the jaws

1 - teeth absent from the anterior portion of the jaws

2 - teeth confined to the anterior part of the jaws

3 - jaws toothless

58. Largest maxillary teeth positioned posteriorly (Kellner 2003):

0 - absent

1 - present

59. Variation in the size of the anterior teeth with the 5th and 6th smaller than the 4th and 7th (Kellner 2003):

0 - absent

1 - present

60. Teeth with a broad and oval base (Kellner 2003):

0 - absent

1 - present

61. Teeth finely serrated (Dalla Vecchia 2009):

0 - absent

1 - present

62. Peg-like teeth (Kellner 2003):

0 - absent

1 - present, 15 less on each side of the jaws

2 - present, more than 15 on each side of the jaws

63. Laterally compressed and triangular teeth (Kellner 2003):

0 - absent

1 - present

AXIAL SKELETON

64. Atlas and axis (Howse 1986):

0 - unfused

1 - fused

65. Notarium (Bennett 1994):

0 - absent

1 - present

66. Postexapophyses on cervical vertebrae (Howse 1986):

0 - absent

1 - present

67. Lateral pneumatic foramen on the centrum of the cervical vertebrae (Kellner 2003):

0 - absent

1 - present

68. Mid-cervical vertebrae (Howse 1986):

0 - short, sub-equal in length

1 - elongated

2 - extremely elongated

69. Cervical ribs on mid-cervical vertebrae (Kellner 2003):

0 - present

1 - absent

70. Neural spines of the mid-cervical vertebrae, height (Howse 1986):

0 - tall

1 - low

2 - extremely reduced or absent

71. Neural spines of the mid-cervical vertebrae, shape (Kellner 2003):

0 - blade-like

1 - spike-like

2 - ridge

72. Number of caudal vertebrae (Unwin 2003):

0 - more than 15

1 - 15 or less

73. Caudal vertebrae with elongated zygapophyses forming rod-like bony processes (Unwin 2003):

0 - absent

1 - present

74. Proximal caudal vertebrae with duplex centra (Bennett 1994):

0 - absent

1 - present

PECTORAL GIRDLE

75. Length of the scapula (modified after Bennett 1994):

0 - subequal or longer than coracoid

1 - scapula shorter than coracoid (1 > sca/cor > 0.80)

2 - substantially shorter than coracoid (sca/cor < 0.80)

76. Proximal surface of scapula (Kellner 2003):

0 - elongated

1 - sub-oval

77. Shape of scapula (Kellner 2003):

0 - elongated

1 - stout, with constructed shaft

78. Coracoidal contact surface with sternum (modified after Kellner 2003):

0 - flattened

1 - oval

79. Coracoidal contact surface with sternum (modified after Kellner 2003):

0 - no developed articulation surface

1 - articulation straight or slightly concave

2 - articulation strongly concave

80. Posterior expansion on articulation surface of the coracoid with the sternum (Kellner 2003):

0 - absent

1 - present

81. Deep coracoidal flange (Kellner 2003):

0 - absent

1 - present

82. Broad tubercle on ventroposterior margin of coracoid (Kellner 2003):

0 - absent

1 - present

83. Cristospine (Kellner 2003):

0 - absent

1 - shallow and elongated

2 - deep and short

FORELIMB

84. Proportional length of the humerus relative to the metacarpal IV (hu/mcIV) (Kellner 2003):

0 - hu/mcIV > 2.50

1 - 1.50 < hu/mcIV < 2.50

2 - 0.40 < hu/mcIV < 1.50

3 - hu/mcIV < 0.40

85. Proportional length of the humerus relative to the femur (hu/fe) (Kellner 2003):

0 - hu/fe < 0.80

1 - 1.4 > hu/fe > 0.80

2 - hu/fe > 1.40

86. Proportional length of the humerus plus ulna relative to the femur plus tibia (hu+ul/fe+ti) (Kellner 2003):

0 - humerus plus ulna about 0.80% or less of femur plus tibia length

(hu+ul/fe+ti < 0.80)

1 - humerus plus ulna larger than 0.80% of femur plus tibia length

(hu+ul/fe+ti > 0.80)

87. Pneumatic foramen on the ventral side of the proximal part of the humerus (Kellner 2003):

0 - absent

1 - present

88. Pneumatic foramen present on dorsal side of the proximal part of the humerus (Bennett 1994):

0 - absent

1 - present

89. Deltopectoral crest of the humerus (modified after Bennett 1994 and Kellner 2003):

0 - reduced, positioned close to the humerus shaft

1 - enlarged, proximally placed, with almost straight proximal margin

2 - enlarged, hatchet shaped, proximally placed

3 - enlarged, hatched shaped, positioned further down the humerus shaft

4 - enlarged, warped

5 - long, proximally placed, curving ventrally

90. Medial (= ulnar) crest of the humerus (Bennett 1994):

0 - absent or reduced

1 - present, directed posteriorly

2 - present, massive, with a developed proximal ridge

91. Distal end of the humerus (Bennett 1989):

0 - oval or D-shaped

1 - subtriangular

92. Proportional length of the ulna relative to the metacarpal IV (ul/mcIV) (Unwin 2003):

0 - ulna 3.6 times longer than metacarpal IV (ul/mcIV > 3.6)

1 - length of ulna between 3.6 and two times the length of metacarpal IV (3.6 > ul/mcIV > 2)

2 - ulna between two times and the same length of metacarpal IV (2 > ul/mcIV > 1)

3 - ulna about the same length or smaller than metacarpal IV (ul/mcIV < 1)

93. Diameter of radius and ulna (modified from Bennett 1994):

0 - subequal

1 - diameter of the radius about half that of the ulna

2 - diameter of the radius less than half that of the ulna

94. Distal syncarpals, shape (Bennett 1994):

0 - irregular

1 - from a rectangular unit

2 - form a triangular unit

95. Pteroid (Kellner 2003):

0 - absent

1 - shorter than half the length of the ulna

2 - longer that half the length of the ulna

96. Metacarpals I – III (Bennett 1994):

0 - articulating with carpus

1 - metacarpal I articulates with carpus, metacarpals II and III reduced

2 - not articulating with carpus

97. Proportional length of the first phalanx of manual digit IV relative to the metacarpal IV (ph1d4/mcIV) (Kellner 2003):

0 - both small and reduced

1 - both enlarged with ph1d4 over four times the length of mcIV (ph1d4/mcIV>4.0)

2 - both enlarged with ph1d4 about or less than two times the length of mcIV (ph1d4/mcIV<2.0) about 2 or smaller

98. Proportional length of the first phalanx of manual digit IV relative to the tibiotarsus (ph1d4/ti) (Kellner 2003):

0 - ph1d4 reduced

1 - ph1d4 elongated and less than twice the length of ti (ph1d4/ti smaller than 2.00)

2 - ph1d4 elongated about or longer than twice the length of ti (ph1d4/ti subequal/larger than 2.00)

99. Proportional length of the second phalanx of manual digit IV relative to the first phalanx of manual digit IV (ph2d4/ph1d4) (Kellner 2003):

0 - both short or absent

1 - elongated with second phalanx about the same size or longer than first (ph2d4/ph1d4 larger than 1.00)

2 - elongated with second phalanx up to 30% shorter than first (ph2d4/ph1d4 between 0.70 - 1.00)

3 - elongated with second phalanx more than 30% shorter than first (ph2d4/ph1d4 smaller than 0.70)

100. Proportional length of the third phalanx of manual digit IV relative to the first phalanx of manual digit IV (ph3d4/ph1d4) (Kellner 2003):

0 - both short or absent

1 - ph3d4 about the same length or larger than ph1d4

2 - ph3d4 shorter than ph1d4

101. Proportional length of the third phalanx of manual digit IV relative to the second phalanx of manual digit IV (ph3d4/ph2d4) (Kellner 2003):

0 - both short or absent

1 - ph3d4 about the same size or longer than ph2d4

2 - ph3d4 shorter than ph2d4

102. Proportional length of the forth phalanx of manual digit IV relative to the first phalanx of manual digit IV (ph4d4/ph1d4) (Kellner 2003):

0 - both short or absent

1 - both elongated, with the forth phalanx the longer than the first (ph4/d4/ph1d4>1.00)

2 - both elongated with the forth phalanx the same length or shorter, but longer than 35% the length of the first (1.00>ph4d4/ph1d4>0.35)

3 - both elongated with the forth phalanx less than 35% the length of the first (ph4d4/ph1d4<0.35)

HINDLIMB

103. Proportional length of the femur relative to the metacarpal IV (fe/mcIV) (Kellner 2003):

0 - femur about twice or longer than metacarpal IV

(fe/mcIV > 2.00)

1 - femur longer but less than twice the length of

metacarpal IV (1.00 < fe/mcIV < 2.00)

2 - femur about the same length or shorter than

metacarpal IV (fe/mcIV < 1.00)

104. Length of metatarsal III (Kellner 2003):

0 - more than 30% of tibia length

1 - less than 30% of tibia length

105. Fifth pedal digit (Kellner 2003):

0 - with four phalanges

1 - with 2 phalanges

2 - with 1 or no phalanx (extremely reduced)

106. Last phalanx of pedal digit V (Wang et al. 2009):

0 - reduced or absent

1 - elongated, straight

2 - elongated, curved

3 - elongated, very curved (boomerang shape)

DATA MATRIX

***Ornithosuchus longidens***

0000000-00 000000---0 0000----00 0000--0-00 0000000000 0000--0000 0000000000 0000000000 0000000000 0000000000 000000

***Herrerasaurus ischigualastensis***

0000000-00 000000---0 0000----00 0000--0-00 0000000000 0000--0000 0000000000 0000000000 0000000000 0000000000 000020

***Anurognathus ammoni***

010001???0 ?????0---0 00?0----0? ?0?0--0-?? ??????0??? 0000--0000 110?0??0?? ?100??0??? ???011??10 000?1011?? ??001?

***Rhamphorhynchus muensteri***

0010100-00 000010---0 0000----00 0000--0-01 1000000110 1010--0000 1000000000 0010000010 0011110020 011?101222 221012

***Pterodactylus antiquus***

0010101000 000010---0 0011001000 1000--0-12 1?0?000??? 1000--0000 1200000111 0100000010 0012110050 021?102122 222020

***Nyctosaurus gracilis***

0010101000 0000?0---0 0010----00 1000--0-0? 110?000121 1000--3000 1001110010 0100000010 0013111030 ?312222222 2?20??

***Pteranodon longiceps***

1020101010 300010---0 001112-000 1101221101 2101000121 1000--3000 1001111010 1101110120 0012111041 1312222222 232020

***Istiodactylus latidens***

0010101100 2???10---0 0?11110?01 21?0--0-01 2?01?00??1 0000--2000 101?1110?0 1???111020 002?1?0141 1?22?????? ??????

***Nurhachius ignaciobritoi***

0010101100 ?000?0---0 001?????01 110??????1 2?????0??? ?000--2000 10101??010 1???1?1020 0?22110?41 ?222?1212? ??21??

***Tropeognathus mesembrinus***

0010101000 3000110200 101?????00 1101001001 2101020121 1001010000 100??????? ?????????? ?????????? ?????????? ??????

***Anhanguera santanae***

0010101010 3000110200 1011100100 1101001001 2101010121 100??10010 1001011010 11??211021 00????0141 1?22?1???? ??????

***Anhanguera blittersdorffi***

0010101010 3000110200 101?????00 1101001001 2101010121 1001010010 100??????? ?????????? ?????????? ?????????? ??????

***Anhanguera piscator***

0010101010 3000110200 1011100100 1101001001 21010?0121 1001010010 1001011010 1101211121 0022110141 1222?1???? ??2120

***Dsungaripterus weii***

2010101000 1101111100 0110----00 1001211101 2111110121 1000--1101 1001111010 010?000??? 0?2210??5? 0311?12122 2?2120

***"Phobetor" parvus***

0010101000 1101111100 0110----00 1001211101 2?1?1?0??? 1000--1101 100??????0 ?????????? ?????????? ???1?????? ??????

***Quetzalcoatlus sp.***

0010101000 3010?14?00 0010----00 100??????1 21???001?? 1000--3000 1001110212 2?0?000020 10?20?1052 0311??2132 2?2?20

***Azhdarcho lancicollis***

?????????? 3????????? ?????????? ?0???????? ?????????? ??????3000 10?1110212 2????????? ??????105? 0????????? ??????

***Zhejiangopterus linhaiensis***

0010101000 3010?0---0 0010----00 1000--0-01 2?1???0??? ?000--3000 10011??212 2?0?000??? 10?200??5? ?31?2?213? ??2???

***Chaoyangopterus zhangi***

1010101??? ?0???????0 00???????? ?????????? ??????0??? 1000--3000 100?010110 0???000??? 00?200???? ?31?2?2122 2321?0

***Shenzhoupterus chaoyangensis***

1010101101 301020---0 001?????0? 2001241101 2?1???0??? 1000--3000 100?01011? ????0??0?? 0??200???? ?31???2122 2321??

***Tupuxuara leonardii***

0010101100 3010213400 001?????01 1001231101 2111130121 1001123000 1001111010 0???000020 0122101052 0311?1213? ??2???

***Thalassodromeus sethi***

0010101100 3010213400 001112-001 1001231101 2111130121 1000--3000 100??????? ?????????? ?????????? ?????????? ??????

***Tupandactylus imperator***

0011101100 3010213301 001??????1 1001221101 2?1??????? ???1133000 100??????? ?????????? ?????????? ?????????? ??????

***Europejara olcadesorum*** **gen. et sp. nov.**

?01??????? ????2????? ?01??????? ????????01 20?????1?1 1101133000 100??????? ?????????? ?????????? ?????????? ??????

***Tapejara wellnhoferi***

0011101100 3010213300 0011111011 1001221101 2011101121 1101133000 100?011010 0?0?000020 01?2101152 03112?21?? ??2120

***Sinopterus dongi***

0011101100 3010?13300 0011100011 1011211101 2?1???1??? ?101143000 100?010110 0???000020 0??2111?52 ?31?2?2122 2321??

***Eopteranodon* *lii***

001110110? ?????13300 00???????1 1????????? ?1???0???? 1?01143000 000??1???? 0?????0??? ???211??5? ?31?2?2122 2?2???

**"*Huaxipterus*" *corollatus***

0011101??? ?????1?310 001??????? ?????????? ?????????? ?101143000 100?01011? ????000??? 0??211??5? ?3????2132 2321??

**"*Huaxipterus*" *benxiensis***

0011101100 ?0???13310 001??????1 10?1211101 2?1???1??? 1101143000 100?01011? ?????????? ???2?????? ?3??2?2122 2?2120

REFERENCES

Andres B, Ji Q (2008) A new pterosaur from the Liaoning Province of China, the phylogeny of the Pterodactyloidea, and the convergence in their cervical vertebrae. Palaeontology 51: 453–469.

Bennett SC (1989) A pteranodontid pterosaur from the Early Cretaceous of Peru, with comments on the relationships of Cretaceous pterosaurs. J. Paleontol. 63: 669–677.

Bennett SC (1994) Taxonomy and systematics of the Late Cretaceous pterosaur *Pteranodon* (Pterosauria, Pterodactyloidea). Occas Pap Mus Nat Hist Univ Kansas 169: 1–70.

Dalla Vecchia FM (2009). Anantomy and systematics of the pterosaur *Carniadactylus* gen. n. *rosenfeldi* (Dalla Vecchia, 1995). Riv Ital Paleontol Strat 115: 159–188.

Howse SCB (1986) On the cervical vertebrae of the Pterodactyloidea (Reptilia: Archosauria). Zool J Linn Soc 88: 307–328.

Kellner AWA (2003) Pterosaur phylogeny and comments on the evolutionary history of the group. In: Buffetaut E, Mazin J-M, eds. Evolution and Palaeobiology of Pterosaurs. Geological Society, London, Special Publications 217: 105–137.

Kellner AWA (2004) New information on the Tapejaridae (Pterosauria, Pterodactyloidea) and discussion of the relationships of this clade. Ameghiniana 41: 521–534.

Martill DM, Naish D (2006) Cranial crest development in the azhdarchoid pterosaur *Tupuxuara*, with a review of the genus and tapejarid monophyly. Palaeontology 49: 925–941.

Swofford DL (2000) PAUP*. Phylogenetic Analysis Using Parsimony (*and other methods). Version 4. Sunderland: Sinauer Associates.

Unwin DM (1995) Preliminary results of a phylogenetic analysis of the Pterosauria (Diapsida: Archosauria). In: Sun A, Wang Y, eds. Beijing: 6th Symposium on Mesozoic Terrestrial Ecosystems and Biota. pp 69–72.

Unwin DM (2003) On the phylogeny and evolutionary history of pterosaurs. In: Buffetaut E, Mazin J-M, eds. Evolution and Palaeobiology of Pterosaurs. Geological Society, London, Special Publications 217: 139–190.

Wang X, Kellner AWA, Zhou Z, Campos DA (2005) Pterosaur diversity and faunal turnover in Cretaceous terrestrial ecosystems in China. Nature 437: 875–879.

Wang X, Kellner AWA, Jiang S, Meng X (2009) An unusual long-tailed pterosaur with elongated neck from western Liaoning of China. An Acad Bras Cienc 81: 793–812.

Wang X, Kellner AWA, Jiang S, Cheng X (2012) New toothed flying reptile from Asia: close similarities between early Cretaceous pterosaur faunas from China and Brazil. Naturwissenschaften 99: 249–257.
